# Proinflammatory Effect of Mesenchymal Stem Cells From Patients With Multiple Sclerosis: Potential Modulation Targets

**DOI:** 10.1212/NXI.0000000000200444

**Published:** 2025-08-28

**Authors:** Radu Tanasescu, Nanci Frakich, Kiranmai Gumireddy, Sonali Majumdar, Sarah Thevathas, Rhodri Jones, Bruno Gran, David Onion, Pryiankara Jayamanna Wickramasinghe, Cherry Chang, Andrew V. kossenkov, Ian Spendlove, Sergio L. Colombo, Louise C. Showe, Cris S. Constantinescu

**Affiliations:** 1 Department of Neurology, Nottingham Centre for MS and Neuroinflammation, Queen's Medical Centre, Nottingham University Hospitals NHS Trust, United Kingdom;; 2 Academic Unit of Mental Health and Clinical Neuroscience, University of Nottingham, United Kingdom;; 3 Biomedical Research Laboratories (BRL), School of Medicine, University of Nottingham, United Kingdom;; 4 Wistar Institute, Philadelphia, PA;; 5 School of Life Sciences, University of Nottingham, United Kingdom;; 6 Department of Haematology, Queen's Medical Centre, Nottingham University Hospitals NHS Trust, United Kingdom;; 7 Academic Clinical Oncology, University of Nottingham, United Kingdom;; 8 Centre for Diabetes, Chronic Diseases, and Ageing, School of Science and Technology, Nottingham Trent University, Nottingham, United Kingdom; and; 9 Cooper Neurological Institute, Cooper Medical School of Rowan University, Camden, NJ.

## Abstract

**Background and Objectives:**

Mesenchymal stem cells (MSCs) represent a potential cellular therapy for multiple sclerosis (MS). It has been suggested that MSCs from patients with MS have lower immunomodulatory properties than those from healthy controls (HCs). The aims of this study were to compare the immunomodulatory abilities of MSCs from patients with MS and HCs against autologous immune cells and to identify potential targets affecting this ability.

**Methods:**

MSCs were obtained by bone marrow aspiration from 5 people with MS and 7 HCs. Autologous peripheral blood mononuclear cells (PBMCs) were stimulated in monoculture or co-culture with MSCs and tested for T-cell expression of IL-17, IFN-γ, and granulocyte-macrophage colony-stimulating factor (GM-CSF) by flow cytometry. Supernatants of monoculture unstimulated MSCs were tested for a panel of cytokines and chemokines using multiplex array or individual ELISA. Gene expression profiling in MSCs was studied using Lexogen QuantSeq RNA-seq platform and subsequent Ingenuity Pathway Analysis.

**Results:**

The MSCs from HCs reduced the proportion of autologous T cells expressing IL-17 (Th17, *p* = 0.046), IFN-γ (Th1, *p* = 0.03), and GM-CSF (*p* = 0.012), whereas MSCs from patients with MS had an opposite effect, increasing both autologous Th17 cells (*p* = 0.01) and GM-CSF–expressing T cells (*p* = 0.03), with no changes in Th1 cell abundance. Unstimulated MSCs from patients with MS produced less IL-10 (*p* = 0.03) and more osteopontin (OPN) (*p* = 0.002) than those from HCs. Gene expression profiling suggested an increase of ADAM28 in the MS MSCs. This was further confirmed at mRNA (by quantitative polymerase chain reaction [qPCR]) and protein (by flow cytometry) levels. Co-cultures of MS MSCs and autologous PBMCs in the presence of natalizumab, a monoclonal antibody that binds α4β1 and blocks its interaction with both ADAM28 and OPN, resulted in a reduction of Th17 cells. In addition, adding IL-10 to the co-cultures abrogated the increase in Th17 cells, potentially interfering with MS MSC inflammatory effect.

**Discussion:**

Our results suggest that blocking ADAM-28 and OPN interaction with cognate receptors or increasing IL-10 reduces the proinflammatory potential of MSCs from people with MS. Similar approaches could be used in clinical MSC treatments.

## Introduction

Multiple sclerosis (MS) is an immune-mediated disease of the CNS characterized clinically by neurologic deficits with relapsing-remitting and progressive patterns and pathologically by neuroinflammation, demyelination, and neuronal loss.^1^ In MS, the balance between inflammatory factors and the cells producing them, such as Th1, Th17, and ThGM cells, and immunoregulatory factors, including anti-inflammatory cytokines and regulatory T cells, is skewed in favor of inflammation.^2^

While significant advances have been made in the treatment of relapsing MS, through immunomodulating and immunosuppressing therapies, the treatment of progressive MS is challenging.^3^ However, therapeutic approaches that target inflammation and that also confer neuroprotection or enhance tissue repair provide future hope for progressive forms of MS.

Treatment with bone marrow–derived mesenchymal stem cells (MSCs), or multipotent mesenchymal stromal cells, could potentially provide a therapeutic approach to autoimmune diseases, including MS.^4^ The observed effects of MSCs may be mediated through modulation of T cells and other immune cells.^5,6^ It has been demonstrated that both murine and human-derived MSCs can ameliorate disease severity in the animal model of MS, experimental autoimmune encephalomyelitis (EAE).^7^ The benefit of MSCs in EAE is attributed to their capacity to downregulate Th1-mediated and Th17-mediated proinflammatory responses^8^ while promoting Treg expansion and responses through a variety of mechanisms, which include, among others, interleukin (IL)-10.^9^

Some studies showing immunomodulatory properties of MSCs have used models of interaction between normal MSCs and normal immune cells, but few show the interaction between endogenous MSCs in patients with MS and their own immune cells. Similarly, most studies investigating the effects of MSCs on EAE have used MSCs transferred from naïve mice to mice with EAE. However, 1 study showed that MSCs from EAE mice, in contrast to MSCs from naïve mice, do not have immunomodulatory properties.^10^ Some MS studies also tend to show a reduced immunomodulatory potential of MSCs from patients. Others show a dissociation of immunomodulatory activity depending on the type of pathogenic cells; for example, MSCs from patients with MS can reduce the inflammatory activity of Th1 but not of Th17 cells. However, such studies again mostly evaluate patients' MSCs interacting with normal immune cells.^11^

This potentially reduced anti-inflammatory ability of MSCs from patients with MS is also reflected in clinical trials. The largest randomized control trial of MSCs in MS did not show an effect of gadolinium-enhancing MS lesions on MRI scans.^12^

In this study, we aimed to compare the immunomodulatory ability of MSCs from patients with progressive MS with MSCs from healthy controls (HCs) on autologous immune cells. In addition, in the event an enhanced proinflammatory or diminished anti-inflammatory activity of MS MSCs was detected, we proposed to identify differentially expressed factors that could be targetable. We planned to select those factors that could be readily neutralized if overexpressed or increased if underexpressed in future pragmatic clinical trials to enhance the therapeutic potential of MS MSCs.

## Methods

### Patients

Patients with secondary progressive MS (SPMS) were recruited from the MS Clinic at the University Hospitals Nottingham Queen's Medical Centre. Healthy control (HC) volunteers without neurologic or medical conditions were recruited from the University and NHS Community. Ethical approval was obtained from East Midlands National Research Ethics Committee (NRES 12/EM/0263). All patients and HCs provided written informed consent, according to the Declaration of Helsinki.

This was a single-center observational study. Each patient's participation was during a short interval between recruitment and harvest of MSCs and blood cells.

Inclusion criteria were men or women with a diagnosis of MS according to 2010 and 2017 McDonald criteria^13,14^ with clinician-determined SPMS^15^ and no evidence of disease activity (clinical relapse or new T2 hyperintense lesion on MRI performed within the past 6 months).

Exclusion criteria included patients who had been taking disease-modifying or immunomodulatory therapy (β-interferon, glatiramer acetate, and systemic glucocorticoids) in the previous 1 month; taking natalizumab (NTZ) or immunosuppressive medication (including but not limited to mitoxantrone, mycophenolate, and tacrolimus) in the previous 3 months, and taking cyclophosphamide in the previous 6 months.

After signing informed consent, on the day of sample collection, consenting participants underwent a brief physical and neurologic examination in the Division of Clinical Neurology.

### Sample Collection

MSCs were collected through bone marrow aspiration from the donor's posterior iliac crest, at the level of the posterior superior iliac spine. The procedures were performed by a qualified hematologist at Nottingham University Hospital (Neurology Clinical Trials Unit).

During the same session, up to 30 mL of peripheral blood was obtained by venipuncture and peripheral blood mononuclear cells (PBMCs) were isolated using Ficoll density centrifugation according to a standard protocol (Histopaque 1077 Sigma-Aldrich, St Louis, MO). Peripheral blood mononuclear cells were frozen at a density of 5–10 × 10^6^ cells/mL, using a cryoprotective freezing medium (90% fetal bovine serum [FBS] + 10% dimethyl sulfoxide [DMSO]) and stored in liquid nitrogen until completion of sample collection.

### MSC Isolation and Expansion

Isolation of MSCs from the bone marrow sample was performed using Ficoll density centrifugation. After 2 washes with the MSC growth medium (MSCGM, Lonza Bullet Kit PT-3001), cells were counted and seeded at a density of 4 × 10^5^ cells/cm^2^. Human MSCs were cultured using the MSCGM and incubated at 37°C with 5% CO_2_ in a humidified incubator for 24 hrs. Then, nonadherent cells were discarded and adherent cells were washed twice with phosphate buffered saline (PBS), followed by addition of a fresh medium, which was changed every 2 days. At approximately 80% confluence, cells were passaged at a seeding density of 5–6 × 10^3^ cells/cm^2^. The cultures were maintained until passages 5–6. At each passage, aliquots of cells were frozen at 1 × 10^6^ cells/mL using the freezing medium (Lonza MSCGM complete media containing 20% heat-inactivated FBS and 10% DMSO), then stored frozen using a controlled-rate freezing process to −80C, and then placed in liquid nitrogen vapor phase. Commercial cell line Lonza MSC (PT-2501) was used for comparison purposes at matching numbers of passages.

### MSC Phenotyping

MSCs were initially identified by the adherence to plastic.^16^ MSC phenotyping was performed using the cell surface marker panel proposed by the International Society for Cellular Therapy for the minimal identification of human MSCs derived from bone marrow.^17^ Using this panel, MSCs were expected to be positive for CD73, CD90, and CD105 but negative for CD34, CD45, CD11b, CD19, and human leukocyte antigen-D related (HLA-DR). MSCs were assessed using the Human MSC Analysis Kit (BD Stemflow-562245). The cells were analyzed by flow cytometry using a LSRII flow cytometer (BD Biosciences) and Kaluza software (V19 Beckman Coulter).

### MSC and Autologous PBMC Co-Culture and Flow Cytometry Analysis

MSCs were thawed, grown in culture for 1 passage, and then used for co-culture studies. Cells were seeded into 24-well plates, at a density of 2.5 × 10^5^ cells per well. Similarly, autologous PBMCs were thawed (on the same day the MSCs were seeded for the study); washed with PBS; and cultured overnight in RPMI 1640 supplemented with 10% FBS, 100 units/mL of penicillin, 0.1 mg/mL of streptomycin, and 2 mM of glutamine (all from Sigma-Aldrich). After overnight incubation, PBMCs were washed with PBS, counted, and seeded on top of the MSC cultures using a MSC/PBMC ratio of 1:4. Additional wells were seeded only with 1 × 10^6^ PBMCs for control purposes. All PBMCs were activated with anti-CD3 and anti-CD28 (both at 1 mg/mL; BD Pharmingen, 567118 and 555725, respectively) for 3 days and then reactivated with the cell stimulation cocktail (cocktail of phorbol 12-myristate 13-acetate (PMA), ionomycin, brefeldin A, and monensin; eBioscience 00-4970-93) for additional 5 hours. PBMCs were then fixed and permeabilized using BD Cytofix/Cytoperm (554714) reagents, followed by staining of surface marker and intracellular cytokines using APC Cy7 anti-CD4 (566913), PE anti-IL-17 (560436), PerCP Cy5.5 anti–granulocyte-macrophage colony-stimulating factor (GM-CSF) (562258, all from BD Pharmingen), and FITC anti–IFN-γ (IM2716U, Beckman Coulter). Live/dead staining was performed using the Live/Dead Fixable Blue Dead Cell Stain Kit (L23105, Invitrogen). The cells were analyzed by flow cytometry using a LSRII flow cytometer (BD Biosciences) and Kaluza software (V19 Beckman Coulter). eFigure 1 shows the gating strategy and fluorescence minus one controls for the flow cytometry experiments.

The effect of the monoclonal antibody NTZ (10 µg/mL) in co-cultured PBMCs with MS MSCs was assessed by flow cytometry analysis for IL-17–positive T cells. For studies with IL-10 (R&D 1064-ILB-010, 20 ng/mL), MS MSCs were pretreated for 24 hours before co-cultures were set or, for comparison, treated with IL-10 at the time of co-culture setting.

### Cytokine Profiling on MS MSCs and HC MSCs

Supernatants from unstimulated MSCs were tested for a panel of cytokines and chemokines using multiplex array or individual ELISA tests. The culture supernatant from 2.5 × 10^5^ cells was collected after 24-hour incubation. The conditioned medium was recovered, centrifuged, aliquoted, and stored at −80 °C until the sample was processed.

The MILLIPLEX Human High Sensitivity T Cell Panel Multiplex Assay (HSTCMAG-28SK, Merck) was used to measure the following cytokines and growth factors: IL-10, IL-12, IL-1β, IL-6, IL-8, TNF-α, and GM-CSF. The MILLIPLEX Human Circulating Cancer Biomarker Panel - Cancer Multiplex Assay (HCCBP1MAG-58K, Merck) was used to measure hepatocyte growth factor and osteopontin (OPN). Transforming growth factor ß was assessed by ELISA (437707, Biolegend).

### Gene Expression Profiling on MS MSCs and HC MSCs

The High-Pure RNA Isolation Kit (Roche, 11828665001) was used to purify RNA from cultured MSCs following manufacturers' instructions. The RNA extraction protocol was performed when cells were 80% confluent (passages 4 to 6).

Gene expression profiling was performed using the QuantSeqV2 kit from Lexogen, targeting the 3′ end of mRNA transcripts. The xCell algorithm^18^ was used to confirm maintenance of MSC signature and lack of significant fibroblastic, osteoblastic, adipocytic, and chondrocytic differentiation. Differentially expressed genes were identified using RNA-seq by expectation-maximization (RSEM)^19^ along with spliced transcripts alignment to a reference (STAR)^20^ for alignment, and all reads within any coding region of a gene were counted to assess gene expression. Raw counts were tested for differential expression using the DESeq2 method. DESeq2-normalized values were used for heatmap visualization of expression differences (genes with at least 10 raw counts in at least 1 sample were considered). Selected relevant genes with significant change in the expression, also as revealed by Ingenuity Pathway Analysis (IPA, Qiagen), were validated by quantitative polymerase chain reaction (qPCR).

cDNA for PCR was synthesized from total RNA using the SuperScript III First-Strand Synthesis kit (Applied Biosystems, Cat. 18080051) following the manufacturer's instructions. qPCR validation was performed using the following TaqMan probe and primer sets: CD70 (Hs00174297_m1), TGFb1 (Hs00171257_m1), ADAM28 (Hs01032761_m1), AMIGO-3 (Hs03055344_s1), and glyceraldehyde 3-phosphate dehydrogenase (GAPDH) (Hs99999905_m1) from Applied Biosystems. All reactions were performed using the Applied Biosystems 7,500 Fast Real Time PCR System. The average of 3 independent assays for each gene and sample was calculated using the ΔΔ threshold cycle method, normalized to the endogenous reference control gene GAPDH, and represented relative to the control sample.

### Analysis of ADAM28 Expression by Flow Cytometry

MSCs were fixed and permeabilized using BD Cytofix/Cytoperm (554714) reagents, followed by staining of ADAM28 (Novus Biologicals, NBP2-67246 PE). The cells were analyzed by flow cytometry using a LSRII flow cytometer (BD Biosciences) and Kaluza software (V19 Beckman Coulter).

### Statistical Analysis

Statistical analysis was performed using GraphPad Prism 7.04 (GraphPad Software, La Jolla, CA). Unpaired *t* tests for normally distributed data and the Mann-Whitney test for data without normal distribution were applied for comparisons. Significance was set as *p* ≤ 0.05.

### Data Availability

Data are available to researchers on request to the corresponding author.

## Results

### Clinical and Demographic Characteristics of Patients With MS and Controls

A total of 15 participants were recruited for the study. Of these, 8 were patients with SPMS (Table 1) and 7 were HC volunteers, matched for age and sex. None of the patients had received immunosuppressive or immunomodulatory drugs or any disease-modifying treatments for 6 months before inclusion in the study. Regarding their ethnicity, 6 of the patients with SPMS were White British (Caucasian), 1 Asian (from the Indian subcontinent), and 1 African British. MSC isolation failed in 2 patients with MS because of technical difficulties (cells failed to grow). One patient withdrew consent between screening and MSC collection. Of the 5 remaining patients, 3 were women and 2 men with a mean age of 53.6 years (range 42–60 years, SD 6.88). The HC group included 5 women and 2 men with a mean age of 43.7 years (range 26–58 years, SD 12.46). The age difference between the patients with MS and HC groups was not significant (*p* = 0.2).

**Table 1:** Characteristics of Patients With SPMS Who Consented for the Study

Patient number	Sex	Disease duration (y since MS diagnosis)	EDSS score	Previous treatment (mo from stopping to MSC collection)
1^a^	**F**	**8**	**5.5**	Methylprednisolone (96)
2^a^	**M**	**10**	**5**	NTZ (12)
3^a^	**F**	**15**	**6.5**	Mycophenolate (6)
4^a^	**M**	**17**	**6.5**	Mitoxantrone (8); tacrolimus (48); NTZ (6)
5^a^	**F**	**11**	**3.5**	None
6^b^	M	18	6.5	None
7^b^	F	17	6.5	NTZ (6)
8^b^	F	15	5.5	Interferon ß1-a (48)

Abbreviations: EDSS = Expanded Disability Status Scale; F = female; M = male; MSC = mesenchymal stem cell; MS = multiple sclerosis; SPMS = secondary progressive MS.

a MSCs from patients 1 to 5 (in bold) were used for experiments.

b MSC isolation failed in patients 6 and 7. Patient 8 withdrew consent before MSC harvesting.

### MSC Phenotyping Results

**Figure 1:**
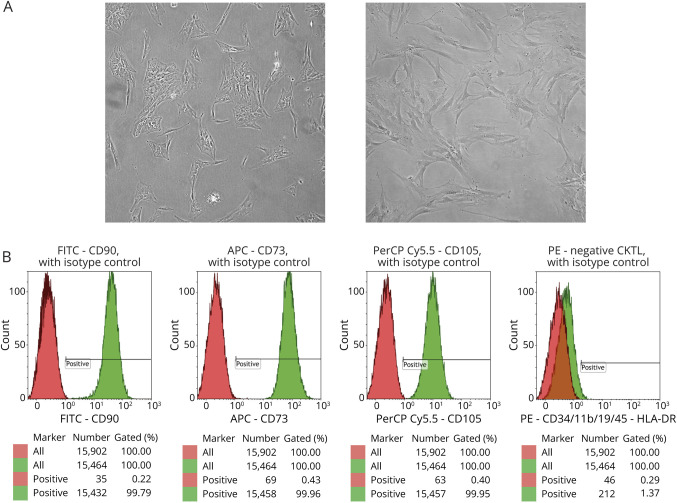
Morphology and Phenotyping of MSCs (A) Representative morphology of MSCs by visible microscopy. Images were obtained under 10X (left) and 40X (right) magnifications. (B) Representative example of flow cytometry analysis showing purity and MSC phenotyping. Preparation usually showed more than 95% expression of CD90^+^, CD73^+^, and CD105+ and less than 2% expression of negative cocktail (CD45^−^, CD34^−^, CD11b-, CD19^−^, and HLA-DR^−^). MSC = mesenchymal stem cell.

MSCs isolated from bone marrow were plastic-adherent when maintained in standard culture conditions and showed a typical spindle shape morphology (Figure 1A). We confirmed the purity and phenotype of all samples at passages 2 and 4 using flow cytometry. MSC population showed >95% expression of CD90, CD73, and CD105 and <2% expression of CD45, CD34, CD11b, CD19, and HLA-DR (CD90^+^, CD73^+^, CD105^+^, CD45^−^, CD34^−^, CD11b^−^, CD19^−^, and HLA-DR^-^) (Figure 1B).

### Co-Culture Results

**Figure 2:**
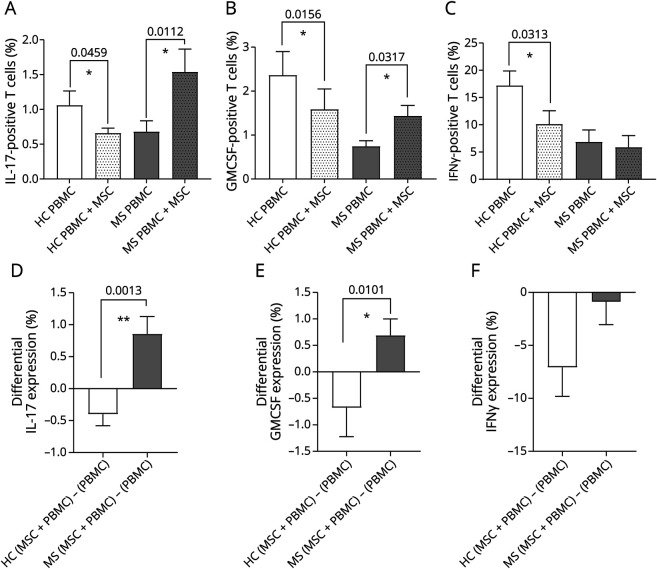
MSCs From Patients With MS Show Deficient Immunomodulatory Effects on Autologous PBMCs The intracellular cytokine expression in PBMCs from patients with MS and HCs, co-cultured in presence/absence of autologous MSCs, was measured by flow cytometry. Panels show the percentage of T cells expressing IL-17 (A), GMCSF (B), and IFN-γ (C) (n = 5 for MS, n = 7 for HC). Panels D, E, and F show, respectively, the differential expression of the cytokines between co-cultured MSCs + PBMCs and PBMCs alone, for both MS and HC groups. MS = multiple sclerosis; MSC = mesenchymal stem cell; PBMC = peripheral blood mononuclear cell

To evaluate the immunomodulatory properties of MSCs, we performed co-culture studies using MSCs and autologous PBMCs. MSCs and PBMCs were thawed for the experiments at intervals that were not different between patients with MS and healthy donors. Activated PBMCs from patients with MS in co-culture with autologous MSCs significantly increased the proportion of T cells expressing IL-17 (Th17, *p* = 0.0112, Figure 2A) and GM-CSF (Th-GM, *p* = 0.03, Figure 2B). The abundance of IFN-γ–expressing T cells (Th1) was unaffected (Figure 2C). By contrast, MSCs from HCs reduced the proportion of autologous T cells expressing IL-17 (Th17, *p* = 0.046, Figure 2A), GM-CSF (*p* = 0.02, Figure 2B), and IFN-γ (Th1, *p* = 0.03, Figure 2C), in co-culture with autologous activated PBMCs. Comparison of the effects of MSCs from patients with MS and those from HCs showed an opposite effect on the differential expression of Th17 and Th-GM cells (Figure 2, D and E), with an observed decrease in the HCs and an increase in pwMS (*p* = 0.001 and *p* = 0.01, respectively). Th1 cells decreased in both pwMS and HCs when co-cultured with autologous MSCs (Figure 2F).

### MSC Cytokine Profiling

Because the immunomodulatory functions of MSCs are believed to be exerted in substantial part by soluble mediators, we investigated the cytokine profile secreted by the MSCs. We applied the Luminex xMAP (multianalyte profiling) technology, which enables the simultaneous detection of multiple secreted proteins. We selected several cytokines and chemokines with immune functions, known to be expressed by MSCs. Supernatants from unstimulated MSCs were tested from phenotyped MSC cultures, at passages between 4 and 6.

**Figure 3:**
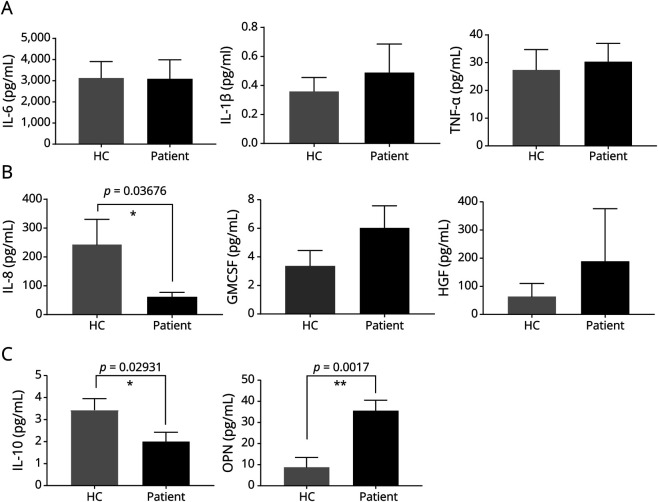
Analysis of Cytokine Expression in MSCs From Patients With MS Data represent the levels of various cytokines measured in the supernatant from cultures of MSCs isolated from patients with MS or healthy controls (HCs). (A) IL-6, IL-1ß, TNF-α; (B) IL-8, GMCSF, hepatocyte growth factor (HGF); (C) IL-10, osteopontin (OPN) (n = 5 for MS, n = 7 for HC). MS = multiple sclerosis; MSC = mesenchymal stem cell.

MSCs from patients with MS showed less production of IL-10 (*p* = 0.03) and more of OPN (*p* = 0.002) than those from HCs (Figure 3C). There were also decreased IL-8 levels in MS MSC supernatants compared with controls (*p* = 0.04, Figure 3B). There were no differences in the expression of IL-6, IL-1β, TNFα, and GM-CSF between HCs and pwMS, despite an insignificant trend for the latter. IL-12 was also measured, but levels were undetectable in all MSCs.

### Differential Gene Expression Analysis and Validation of Selected Targets

**Figure 4:**
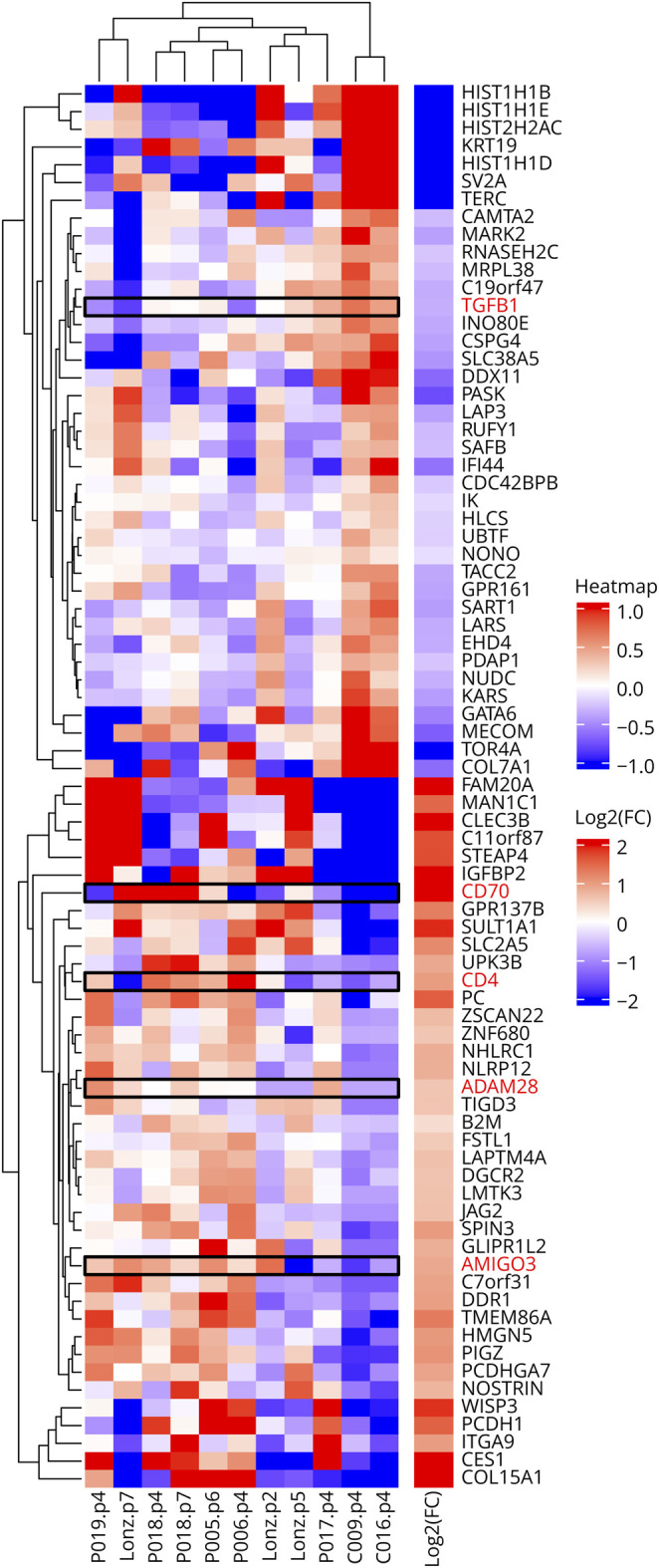
Global Analysis of Gene Expression by RNA-Seq Analysis Heatmap shows differential gene expression across pwMS, Lonza cell line, and HCs. IPA-identified genes considered for further validation and analysis are shown with a black box. CD4 was not validated further (mentioned in text).

RNA-seq assessment of global gene expression was compared across MSCs from pwMS, HCs, and a commercial source, Lonza. Sufficient RNA from 2 HCs was available for these experiments. Because there was significant similarity of HC MSCs with Lonza MSCs, these controls were analyzed separately and pooled for differential expression analysis. From these analyses, 672 genes were identified from the comparison against HCs and 639 against the combination of HC + Lonza controls. Figure 4 shows the heatmap and presents the results obtained using an false discovery rate (FDR) cutoff of 15%. Considering that, in a clinical pragmatic MSC treatment situation, the interaction between MSCs and T cells should be modulated to increase their immunomodulatory potential and decrease proinflammatory effects, we focused on genes whose products could be directly targeted during the interaction of MSCs with T cells, such as surface molecules or secreted cytokines that can be blocked with antibodies. IPA (eTable 1) identified several genes of interest that were potential targets and were prominent in several significant functional categories and 1 network (Figure 4).

The genes of interest included ADAM28, CD70, AMIGO3 (all increased in MS MSCs), and TGF-ß1 (decreased in MS MSCs). ADAM28, a putative proinflammatory molecule, has multiple effects on the immune system, cell migration, and cancer^21^; CD70, a possible MSC marker, was also ascribed proinflammatory effects^22^; and AMIGO-3 plays a possible role in preventing re-myelination.^23^ We found CD4, which can be present on MSCs,^24^ to be expressed at higher levels in MS MSCs, but we decided not to validate this observation, to avoid potential effects on the T cells during this interaction that would mask MSC effects (Figure 4, black-boxed genes).

As expected, using the xCell algorithm,^18^ we found MSC-specific gene signatures to be overwhelmingly represented in the MSC cultures used for PBMC interaction experiments. We did not observe any stable significant enrichment of fibroblast, osteoblast, chondrocyte, preadipocyte, or adipocyte signatures (data not shown).

**Figure 5:**
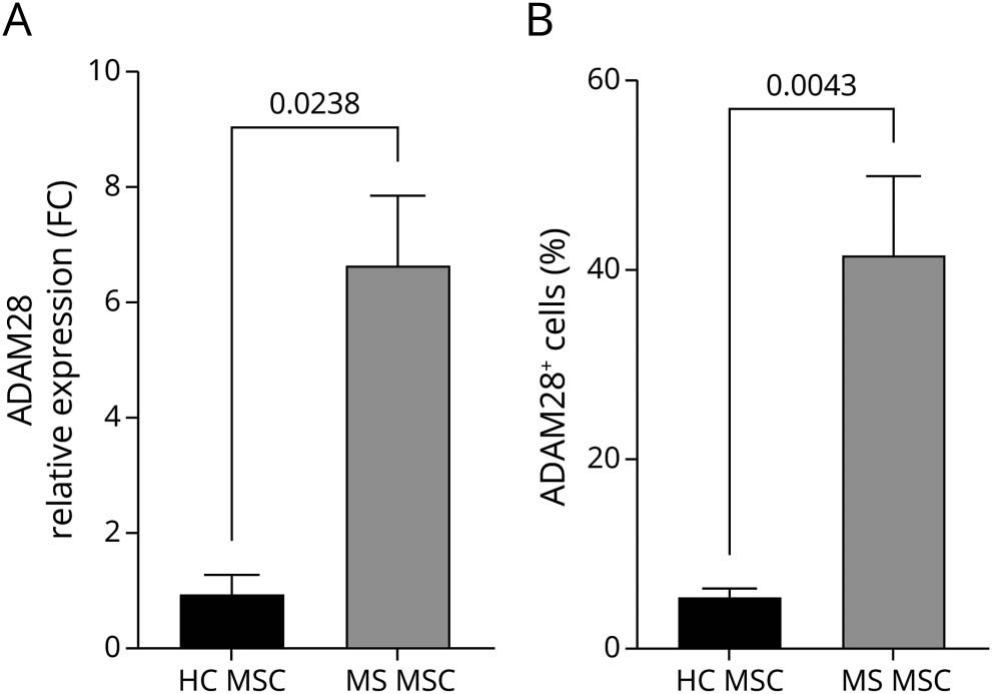
Assessment of ADAM28 Gene Expression on MS MSCs (A) mRNA expression assessed by RT-qPCR. Data are presented normalized to housekeeping and relative to HC MSCs. (B) Protein abundance assessed by flow cytometry on MSCs and presented as percentages of ADAM28+ MSCs (n = 4). HC = healthy control; MSC = mesenchymal stem cell

To validate the IPA results, the relative abundance of the candidate genes was determined by RT-qPCR. Only the mRNA for ADAM28 showed significant upregulation by PCR (Figure 5A and eFigure 2). ADAM28 protein abundance was also assessed by flow cytometry analysis of MSCs from pwMS and HCs. The assessment confirmed that a higher proportion of MSCs from pwMS are positive for ADAM28 protein, representing a significant 7-fold increase over controls (Figure 5B). To determine whether prototypical inflammatory factors increased ADAM28 expression in MSCs, we treated MSCs from pwMS and HCs with IFN-γ and showed that it increased the percentage of ADAM28-positive MSCs from both pwMS and HCs (Figure 5; eFigure3).

### Effect of NTZ on ADAM28 Expression in MS MSCs

**Figure 6:**
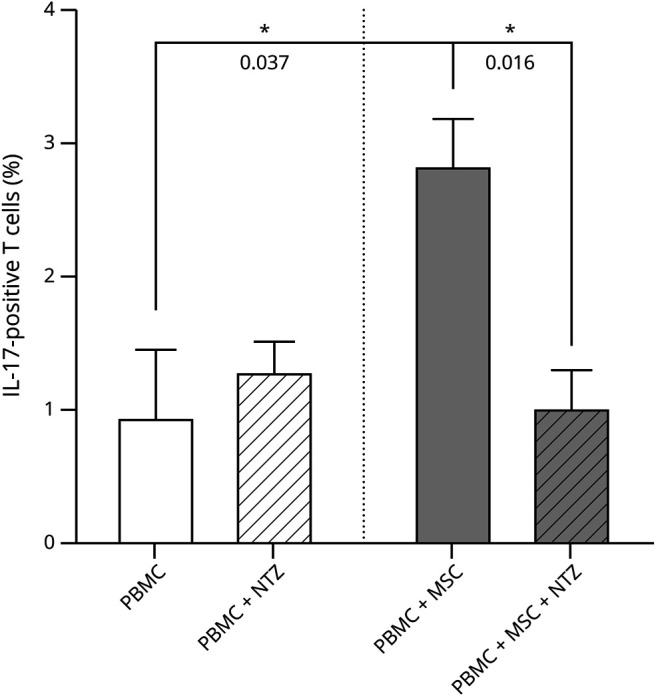
Effect of NTZ on MS MSC Immunomodulation IL-17 + T cells were measured using flow cytometry in PBMCs or in autologous MSC co-cultures isolated from patients with MS. Treatment with NTZ (10 ug/mL) significantly affected immunomodulation in the co-cultures. *Significant difference and number indicate the *p* value observed. MS = multiple sclerosis; MSC = mesenchymal stem cell; PBMC = peripheral blood mononuclear cell.

ADAM28 is known to interact with the integrin VLA-4, and so is OPN, another proinflammatory molecule we found to be upregulated in MS MSCs.^25-27^ These interactions have biological effects relevant to MS. Natalizumab, a monoclonal antibody against VLA-4 that binds it and blocks interactions with its ligands, is used as a highly effective treatment of MS. Thus, we investigated whether a monoclonal antibody against VLA-4 can block the ability of MS MSCs to increase IL-17–producing cells in the interaction with autologous PBMCs. We demonstrate that, indeed, co-culturing MSCs and PBMCs from the same patients with MS in the presence of a monoclonal antibody against VLA-4 abrogates the increase in Th17 cells induced by the interaction between MSCs and PBMCs from pwMS (Figure 6).

### Effects of IL-10 on MS MSCs

**Figure 7:**
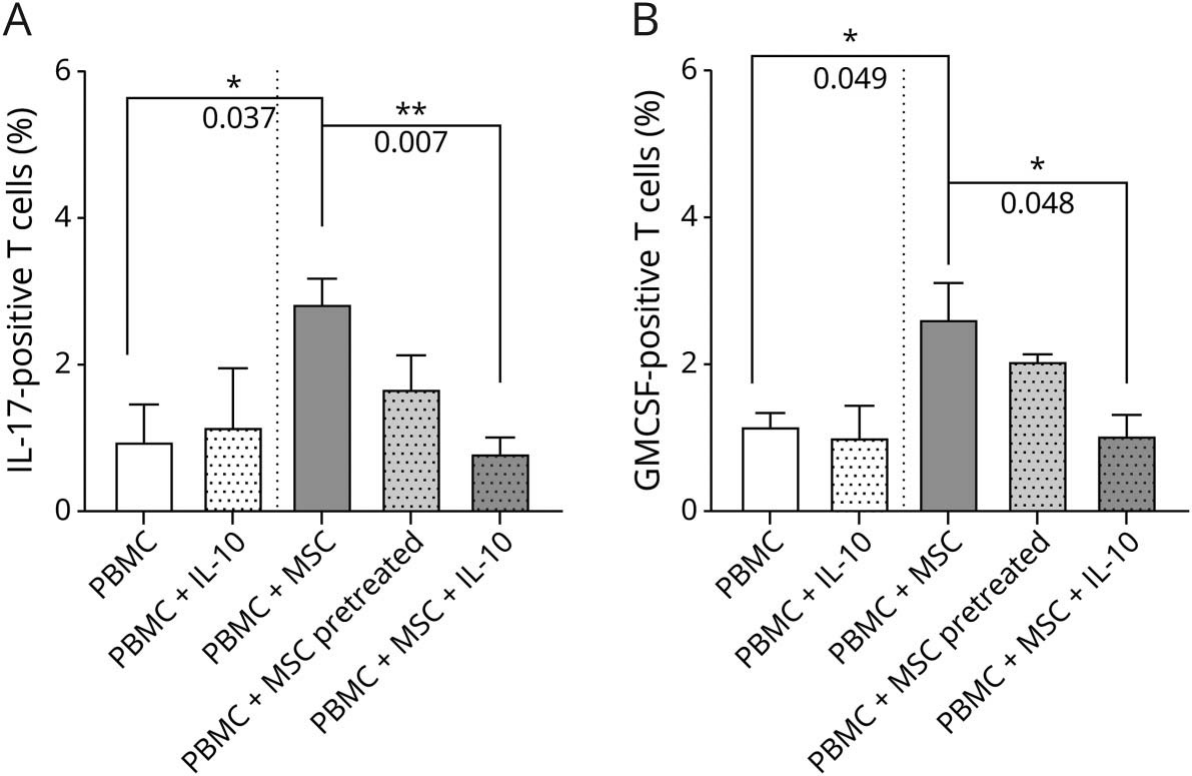
Rescue of MSC Immunomodulatory Activity in Patients With MS by Interleukin-10 Co-culture of patient MSCs and autologous PBMCs was evaluated by flow cytometry in presence of 20 ng/mL of IL-10. (A) IL-17+ T-cell percentage in the co-cultures was significantly reverted in the presence of IL-10, an effect that was not significant when MSCs were pretreated with the cytokine. (B) GMCSF T-cell percentage in the co-cultures was significantly reverted in the presence of IL-10, but this was not significant when pretreated with the cytokine. MS = multiple sclerosis; MSC = mesenchymal stem cell; PBMC = peripheral blood mononuclear cell.

We found that MSCs from patients with MS produce less IL-10 (Figure 3C). We then tested whether adding IL-10 to MSC-PBMC co-cultures would reduce the ability to induce IL-17–positive or GM-CSF–positive cells. We show that pretreating MSCs with IL-10 does reduce the proportion of IL-17–positive or GM-CSF–positive cells (Figure 7, A and B), indicating another potential approach to reducing the proinflammatory potential of MSCs from patients with MS.

## Discussion

Theoretically, MSCs have the potential to target the 2 cardinal pathologic mechanisms of MS, inflammation and neurodegeneration. To date, information on the effect of these cells in vitro and in vivo in MS is incomplete. However, some studies suggest that both immunomodulatory and neuroprotective effects of MSCs from pwMS are not as powerful as those from donors without MS. Some studies indicate that MSCs can modulate Th1 but not Th17 cells.^11,28^ This may also apply to properties of MSCs from patients with other immune-mediated inflammatory diseases as well.^29^ The effects of MSCs on T cells that express GM-CSF, some of which are designated Th-GM, are not well known. The reduced immunomodulatory capacity may be due to reduced ability of MS MSCs to produce immunoregulatory factors, reduced ability of immune cells to undergo regulation, or both. This has been shown, for example, for regulatory T cells (Treg), which have reduced suppressive potential in MS.^30^ Therefore, although MSC treatment of MS can increase the proportion of CD4^+^CD25^+^ T cells,^31^ the regulatory capacity of these cells may be reduced. Considering that both MSCs and immune cells from pwMS have been exposed to an inflammatory milieu, it is important to investigate these cells in autologous interactions. It is also important to compare these effects with those of MSCs from healthy donors on autologous immune cells because differences can provide important clues to modalities of overcoming the proinflammatory potential of MSCs from patients with MS.

In these studies, we show that MSCs from pwMS, when interacting with autologous immune cells, not only lack immunomodulatory activity on these immune cells but also enhance their inflammatory potential, by increasing the proportion of Th17 and Th-GM cells, and not reducing the proportion of Th1 cells. This is contrast to MSCs from non-MS HCs, which have an immunomodulatory effect on autologous Th17 and Th-GM cells but also lack significant effects on the proportion of Th1 cells. The lack of the immunomodulatory potential in MS MSCs that is traditionally attributed to MSCs may be explained by an intrinsic deficit in immune regulation, as supported by the evidence of a numeric and/or functional Treg deficit and by the coexistence with other autoimmune disorders. Alternatively, long exposure to an inflammatory milieu may cause the MS MSCs to lose immunomodulatory properties and become proinflammatory.

This has implications for autologous MSC treatment of autoimmune diseases including MS. Possibly, the positive effects of MSCs on inflammation and tissue repair are counteracted or diminished by these paradoxical proinflammatory effects. This may explain the negative or only modest results of clinical trials of MSCs in MS. This study, however, also identifies some possible ways of abrogating these proinflammatory effects.

Immunomodulatory activity of MSCs from patients with MS can be restored by blocking proinflammatory factors and enhancing anti-inflammatory factors in vitro, before the transfer of MSCs. In experimental settings, human MSCs engineered to secrete immunomodulatory factors, e.g., IFN-β, have improved outcomes of MSC treatment in the EAE model.^32^

A recent open-label trial explores the effects of MSCs from patients with MS engineered to be enriched in neurotrophic factors and administered intrathecally.^33^ The results of the trial are promising. An approach whereby MSCs undergo enrichment of neurotrophic effects while being targeted directly to the CNS may be more successful. A very recent phase II randomized, double-blind, placebo-controlled clinical trial in progressive MS using intrathecally administered MSC-neural progenitors derived from bone marrow MSCs demonstrated increased MMP9 and decreased CCL2 levels in the CSF after treatment and potential benefit in a subgroup of pwMS.^34^ Of note, the measured outcomes of such approaches have been primarily based on the neuroprotective, rather than immunomodulatory, effects of MSCs. Our objective was to identify modalities of treating MSCs without much in vitro manipulation or genetic modification, which would make them utilizable for pragmatic larger clinical trial settings involving many centers with standard laboratory capacity. In addition, IV rather than intrathecal administration would be preferable at equally good likelihoods of success.

In our cytokine expression study, we noted a deficit of IL-10 in supernatants of MSCs from patients with MS. Addition of IL-10 abrogated the proinflammatory effect of MS MSCs and restored their immunomodulatory potential. Recently, MSCs overexpressing IL-10 showed immunosuppressive properties and reduced inflammation in experimental airway inflammation.^35^ Clinical grade IL-10 reduced inflammation in inflammatory bowel disease trials.^36^ It is, therefore, conceivable that expanding and transferring MSCs in the presence of an IL-10–enriched medium could restore the therapeutic qualities of MSCs from pwMS.

Another cytokine dysregulated in MS MSCs in our study was OPN. This proinflammatory cytokine has multiple effects that are relevant to MS.^37^ It is elevated in plasma and CSF of patients with MS,^38^ and its CSF levels were used as outcome measures in progressive MS trials.^39,40^ OPN binds the integrin VLA4 that is the target of the monoclonal antibody NTZ, a high-efficacy MS treatment. NTZ was shown to reduce expression of OPN in MS and can prevent OPN effects by preventing its binding to VLA4.

In our study, we demonstrated that ADAM28 has an increased expression in MSCs from pwMS compared with controls. This was validated by quantitative qPCR and at the protein level. We showed that proinflammatory stimuli, e.g., IFN-γ, increase expression of ADAM28 in human primary MSCs and in the Lonza cell line (not shown). ADAM28 encodes a disintegrin and metalloprotease with multiple functions that are increasingly explored. It enhances effector immune cell function, being expressed in lymphocytes, and it mediates cell infiltration and migration. It is also expressed in mesenchymal cells, particularly at the epithelial-mesenchymal transformation that occurs in cancer.^41^

We show here that NTZ reduces the Th17-inducing and Th-GM–inducing effect of MS MSCs in interaction with autologous PBMCs. Investigating the detailed mechanism is beyond the scope of this study, but correction of dysregulated OPN or ADAM28 or their proinflammatory effects on binding VLA4 is an example of a possible mechanism. NTZ is an approved, effective treatment of MS. Its main mechanism of action is by blocking migration of inflammatory cells into the CNS, but other mechanisms relying on binding and blocking VLA4 have been suggested.^42,43^ Thus, it is conceivable that the presence of NTZ during the interactions of MSCs and T cells prevents engagement of VLA4 by ADAM28 and reduces its proinflammatory effect. Of note, NTZ is only an example of how MSC potential can be modulated in a practical trial setting, given that NTZ is an approved and widely used treatment of MS. This approach has the potential of identifying more and possibly better targets.

Our study suggests that transplanting autologous MSCs that have been cultured in the presence of NTZ and/or IL-10 or infused alongside NTZ and/or IL-10 may make them more immunomodulatory and enhance their therapeutic potential.

Treating MSCs with NTZ may, in principle, reduce their CNS migration potential,^44,45^ although they may only express β1 integrin and not α4, the other component of VLA4. Immunomodulatory effects of MSCs on neuroinflammation may not be entirely dependent on CNS migration as suggested by studies showing that this effect although most intravenously transferred MSCs do not reach the CNS, being retained mainly in the liver.^46,47^ On the contrary, for their tissue repair and neuroprotection, MSCs may need to reach damaged tissue. For this, whether they are treated with a substance that reduces the β1 integrin interactions, their ability to reach the CNS after IV infusion is low. Therefore, options such as intrathecal transfer provide hope for future MSC transfer treatments, and preliminary results suggest stronger neuroprotective effects with this approach. It is thus hoped that MSC treatment with compounds that enhance their immunomodulatory potentials by intrathecal routes would fulfil both main objectives of MSC treatment: immunomodulation and neuroprotection/repair.

Strengths of our study are the evaluation of MSCs and peripheral blood immune cells from the same individual and the comparison with MSCs and peripheral blood immune cells from non-MS controls. This is among the first studies performed with this design. Although the main aim was to confirm, in this interaction of cells from the same individual and in direct comparison with normal cells, the deficiency of immunomodulatory effects of MS MSCs suggested by previous studies, we also observed effects that are proinflammatory, more than just deficient immunomodulation. We were also able to discover some targetable molecules that may mediate this proinflammatory effect. Another strength is the applicability to clinical interventions using MSCs in MS. Not only do the in vitro interactions of MSCs and peripheral blood immune cells of the same donor more closely reflect the in vivo interactions, but their treatment with available, clinical-grade compounds such as human recombinant IL-10 or NTZ provides the opportunity to suppress the proinflammatory effects of MS MSCs and their interaction with immune cells, without in vitro genetic manipulation.

Our study has some limitations. The number of donors is relatively small, and the quantity of PBMCs was limited, restricting the number of experiments possible. In this small sample, there were age differences between the healthy volunteers and the patients with MS, and thus, the results may be attributed to more MSC and immune senescence in the slightly older MS group. However, the age differences were small and not statistically significant and there were no patients who could be classified as elderly as defined for MSC purposes.^48^ There is evidence of premature MSC^49^ and immune aging in MS,^50^ but matching for this instead of chronological aging is not feasible before a study like ours. However, we cannot eliminate the possibility that there may be some effects related to (biological) age.

The effect of the interaction on Treg cells was not investigated, because of restricted number of cells. The molecules chosen for the targeted study were not selected from the basic differential gene expression between the patient and control classes but from the IPA functional analysis where they were overwhelmingly represented in a number of functional groups and because they were biologically justifiable as being potentially important and targetable. Our corroborating results from a variety of different assays that examined protein levels and functional properties support this approach. Other targets from this study and from future studies may be tested using this approach.

In conclusion, we provide evidence for a proinflammatory effect of MSCs from pwMS on autologous immune cells and further show that targeting some molecules that are differentially expressed between pwMS and non-MS controls can counteract this proinflammatory effect. These observations can lead to the development of modalities for improving outcomes of MSC treatment of MS.

## Glossary

DMSO: dimethyl sulfoxide
EAE: experimental autoimmune encephalomyelitis
FBS: fetal bovine serum
FDR: false discovery rate
GAPDH: Glyceraldehyde 3-phosphate dehydrogenase
GM-CSF: Granulocyte-Macrophage Colony-Stimulating Factor
HC: healthy control
HLA-DR: Human leukocyte antigen-D related
IPA: Ingenuity Pathway Analysis
MS: multiple sclerosis
MSC: mesenchymal stem cell
NTZ: natalizumab
OPN: osteopontin
PBMC: peripheral blood mononuclear cell
PBS: Phosphate Buffered Saline
qPCR: quantitative polymerase chain reaction
RSEM: RNA-Seq by Expectation-Maximization
SPMS: secondary progressive MS
STAR: Spliced Transcripts Alignment to a Reference
